# Evaluating Knowledge Outcomes of the Age-Friendly Health Systems and 4Ms Module in Medical Education: A Comparative Study of YouTube vs. Virtual Reality Platforms

**DOI:** 10.7759/cureus.104394

**Published:** 2026-02-27

**Authors:** Winnie Chen, Michelle T Ngo, Sweta Tewary

**Affiliations:** 1 Osteopathic Medicine, Nova Southeastern University Dr. Kiran C. Patel College of Osteopathic Medicine, Davie, USA; 2 Geriatrics, Jessie Trice Community Health System, Miami, USA

**Keywords:** age friendly health systems, alzheimer’s dementia, digital learning, geriatric education, innovation, simulation training, virtual reality, virtual reality in medical education, youtube

## Abstract

This study evaluated and compared the effectiveness of Virtual Reality (VR) versus YouTube-based training in teaching the 4Ms framework: What Matters, Medication, Mentation, and Mobility, within a geriatric medicine course for second-year medical students. Pre- and post-training surveys were administered via REDCap, and matched sample t-tests and Cohen’s d effect size were conducted. VR training resulted in a mean knowledge increase of 0.55 (2.49 to 3.04, N=90), while YouTube training showed a 0.53 increase (2.58 to 3.11, N=76). Both showed statistically significant gains (p <0.001), with YouTube having a larger t-value (-7.39 vs. -6.41), but VR demonstrating a greater effect size (Cohen’s d = 0.82 vs. 0.62). However, an independent samples t-test revealed no significance in knowledge gains between groups (p = 0.80). These findings suggest that both modalities effectively enhance knowledge, with VR demonstrating a stronger effect size, likely due to its interactive effect. This highlights its potential use in medical education on geriatrics.

## Introduction

Virtual reality (VR) platforms are popular due to their ability to provide an immersive, hands-on learning experience resembling an actual medical practice or care setting [1]. VR promotes active participation, potentially enhancing memory retention and engagement [2]. While VR education can stimulate a clinic setting and provide students with learning experiences being risk-free for potential patients, this platform also has its limitations of lack of VR expertise from medical education faculty and learners [3]. While VR has traditionally been used in the surgical field to replicate cases, medical schools are integrating VR beyond procedural training, such as in the preclinical/didactic curriculum [2]. For example, VR allows students to go through cases that mimic real-life patient encounters, from taking medical histories, performing physical exams, making diagnoses, and providing treatment plans [4]. Additionally, by interacting with simulated patients who can express varying emotions and complexity, this allows students to further develop critical skills in communication, critical thinking, and clinical decision-making [4]. VR is beneficial in dementia training as it allows students to go at their own pace, which can be harder to otherwise simulate [2].

YouTube (YT) is a free and available social media platform that allows for unorthodox teaching and learning methods through video format [3,5]. YT is a digital tool that is often used in the academic setting post COVID-19 [6]. YT was used to allow students to access content at their own convenience through self-paced learning, which improves academic performance [3,7]. Additionally, YT improves confidence, satisfaction, and methods of learning in students [5]. However, a limitation of using YT as an educational tool can be the lack of student engagement and usage confirmation [5].

The John A. Hartford foundation in collaboration with the Institute for Healthcare Improvement (IHI), the American Hospital Association (AHA), and the Catholic Health Association of the United States (CHA) developed Age Friendly Health System (AFHS) initiative that pushed a movement of improving health care for older adults through an evidence-based framework [8]. The AFHS approach revolves around what matters to older adults and their caregivers while emphasizing evidence-based, low-risk, patient-centered, and coordinated care [9]. The framework used to guide care for older adults and improve quality of life involves the 4Ms domains [9]. The 4Ms of AFHS are What Matters Most, Medication, Mentation, and Mobility [8]. These domains help improve geriatric assessments by identifying patient goals and values, reconciling medications, addressing mental and cognitive health, and ensuring movement safety precautions [10].

AFHS has been implemented across various practice settings, such as hospitals, primary care, and skilled nursing facilities, by embedding the 4Ms framework (What Matters, Medications, Mentation, and Mobility) into routine care. The 4Ms framework has been incorporated into Electronic Health Record (EHR) templates, performance reviews, and staff training across hospital and primary care settings [11,12]. Whereas in skilled nursing facilities, the 4Ms framework has been applied in interprofessional workgroups and national learning collaboratives [13]. Such efforts have shown great results in patient satisfaction, reduced inappropriate Beers Criteria medication orders, and improved ambulation distances after 4Ms adherence rose from 81% to 100% over the past five years [14].

Additionally, AFHS can be implemented on an interprofessional level of a healthcare professional team. It is recommended by the American Geriatrics Society that early introduction of AFHS concepts in medical school ensures that graduating physicians are prepared to deliver age-friendly care [15]. This can be done via modalities such as virtual reality through a case-based simulation.

In our previous work with the geriatric department of Nova Southeastern University, we discussed the development, evaluation, and implementation of a case-based training module highlighting Age Friendly Health System (AFHS) and the 4Ms (What Matters, Medication, Mentation, and Mobility) in geriatric care through a virtual reality platform [16]. The study highlighted knowledge gained among medical students in understanding AFHS during a geriatric course for second-year medical students, and virtual reality was helpful in understanding AFHS and 4Ms through a case-based scenario for AFHS. However, the low sample size limits the generalizability of the results. Additionally, due to time constraints, many students preferred a YT version which was not previously analyzed. Our current study addresses this gap and 1) compares and evaluates knowledge gain before and after implementation of a case-based scenario through a VR platform and 2) the difference in knowledge gain between two different education platforms, VR and YT.

## Materials and methods

Study design and participants

We used a pre-post study design to compare the effectiveness of VR vs YT in introducing the AFHS and 4Ms to second-year medical students. This initiative was part of the geriatric curriculum during the second year. Participants in the study were selected through purposive sampling within the second-year medical students enrolled in the geriatric medicine course. Although all students were given the option of learning the case-based scenario via a YT video or a VR session, the inclusion criterion to participate in the evaluation was voluntary. Data was collected in March 2024 while the lecture was delivered as part of the geriatric course of the curriculum. Data collection included completion of evaluation surveys with 21 questions survey administered through REDCap, a mature and secure program for building and managing online surveys and databases that ensured anonymity.

Data collection, instrument, and evaluation

The survey included multiple-choice questions on demographics such as age, gender, race/ethnicity, veteran status, educational background, and disadvantaged status (See Appendix 1). Content-based questions assessed self-reported perceived knowledge of the topic before and after the training session and perceived likelihood of applying learned concepts in clinical practice on a 4-point Likert scale (None = 1, Low = 2, Moderate = 3, High = 4). No objective standardized knowledge examination was administered. Data was analyzed using SPSS v29 (IBM Corp., Armonk, NY, USA) through descriptive statistics using frequencies and percentages as well as inferential statistics using matched sample t-test and independent sample t-test. The level of statistical significance was set at p < 0.001 in order to reduce the risk of a Type I error. Effect sizes were also calculated using Cohen’s d to measure the magnitude of observed change.

Detailed methods: case study

This case-based scenario was used in both VR and YT platforms within a one-hour training session. The case presents an 87-year-old female named Millie with a past medical history of hypertension, diabetes mellitus, diabetic peripheral neuropathy, urinary incontinence, chronic kidney disease, osteoarthritis, and macular degeneration who fell while going to the bathroom at night. Students first meet Millie at the hospital setting and perform a medication reconciliation. Then, students perform a mentation assessment using screening tools (PHQ-2 and Mini-Cog) to evaluate dementia and depression. Once Millie is ready for discharge to the skilled nursing facility, the students discuss advance care directives with her caregiver, reviewing “What Matters”. Once Millie arrives at the nursing facility, students perform the fall risk assessment of Timed Up and Go (TUG) while answering an interactive quiz to identify fall risk hazards. This case-based scenario addresses each component of the 4Ms.

Ethical considerations

The study was a quality improvement project and nonhuman subject research; thus, IRB approval was waived due to the nature of the study (IRB #2025-223). The study’s protocol does not require IRB review or approval as it does not fall within IRB’s jurisdiction based on 45 CFR 46.102. For IRB purposes, the protocol has been classified as “Research outside the purview of the IRB”. However, this study may still be classified as “research” for academic purposes or for other regulations pertaining to educational records (FERPA) and/or protected health information (HIPAA). The intent of the study was program evaluation, and participation was voluntary. The feedback was requested at the end of the course, and the responses did not impact the participants' grades. All data was de-identified prior to analysis. Confidentiality was maintained by securing information in RedCap with data access limited to the evaluation team.

## Results

Descriptive statistics

The training, including VR and YT versions of education content of AFHS and 4Ms, was completed by 193 participants. However, the pre-post assessments were completed by 166 students with a response rate of 86%. All participants were third-year medical students with the majority between 20 and 29 years old. Of the 193 participants, 71 (37%) were male, 107 (55%) were female, and 15 (8%) did not report. The majority identified themselves as White (n=103, 53%) and were non-Hispanic/Latino (n=152, 79%) (See Table 1).

**Table 1:** Demographics (n = 193)

Characteristics	# of students	% of students
Age		
19 and under	1	1%
20-29	170	88%
30-39	10	5%
60 and over	1	1%
Not reported	11	6%
Gender		
Male	71	37%
Female	107	55%
Not reported	15	8%
Race		
White	103	53%
African American	5	3%
Asian	54	28%
Mixed - selected more than 1 race	7	4%
Not reported	24	12%
Ethnicity		
Hispanic/Latino	26	13%
Not Hispanic/Latino	152	79%
Not reported	15	8%

Knowledge gain pre- and post-training

We used a matched sample t-test to evaluate the pre-post feedback of the knowledge surveys for both groups. For the VR group, a matched sample t-test showed a significant increase in scores from pretest (M = 2.49, SD = 0.86) to post-test (M = 3.04, SD = 0.92); t(-6.41), p < 0.001, d = 0.82 (See Table 2).  Similarly, the YouTube group also showed a significant increase in knowledge with pretest scores (M = 2.58, SD = 0.75) rising to post-test scores (M = 3.11, SD = 0.66; t(-7.39), p < 0.001, d = 0.62 (See Table 2).

**Table 2:** T-Tests of General Knowledge Pre- and Post-Training

Group	Pre-M (SD)	Post-M (SD)	t	df	p	d
VR (n = 90)	2.49 (0.86)	3.04 (0.92)	-6.41	89	< .001	0.82
YouTube (n = 76)	2.58 (0.75)	3.11 (0.66)	-7.39	75	< .001	0.62

Independent samples t-test

An independent samples t-test was performed to determine whether YouTube or VR showed greater overall knowledge gains over the other. The VR group achieved a mean increase of 0.55 (SD = 0.96), whereas the YouTube group achieved a comparable mean increase of 0.53 (SD = 0.77) (See Table 3). The comparison between these two groups indicated no statistically significant difference, t(-0.26), p = 0.80, d = 0.04.

**Table 3:** Independent Samples T-Test

Group	Mean Gain (SD)	95% CI of Difference	t	df	p	d
VR (n = 90)	0.55 (0.96)	-0.26, 0.20	-0.26	164	0.799	0.04
YouTube (n = 76)	0.53 (0.77)	-0.25, 0.19	-0.26	162.08	0.795	

## Discussion

The purpose of our study was twofold: 1) to evaluate if virtual learning can improve knowledge on AFHS and 4Ms, and 2) compare knowledge change between different virtual platforms. We evaluated knowledge before and after the training through both VR and YT platforms. First, we evaluated if there was a difference in knowledge before and after the program in each group. Second, we compared if knowledge gained through VR mode is different from that of YouTube mode.

The matched sample t-test indicated that the training increased knowledge in both VR and YT groups. However, these differences in knowledge post-training may be biased due to the social desirability of responding to the class instructor and self-reported responses. Survey fatigue and participants skipping questions on the knowledge survey were among others. Several participants did not complete the survey or skipped questions and were excluded from the analysis, limiting generalizability. While the results demonstrated that both learning platforms were effective in improving self-reported knowledge, this study did not include a didactic comparison group. Therefore, conclusions regarding the comparative effectiveness of virtual versus classroom teaching cannot be drawn. VR also showed a larger effect size compared to YT. However, any advantages regarding engagement or retention are not conclusive as the between-group comparisons were not statistically significant.

Current research evidence evaluating VR as a modality for teaching AFHS and the 4Ms framework remains limited, as only a few published studies address this learning modality. Our previous study highlighted similar results of significant knowledge improvement in pre-post training, but with a smaller sample [16]. However, the study was not able to capture the differences in learning between YouTube and VR formats. Our second research question in this study addressed the prior limitation by evaluating the differences in the two learning platforms through an independent sample t-test and found that there is no difference in learning between both formats. Results suggest that knowledge learned is not impacted by the different platforms that the information is delivered on, but rather the content itself. These results also suggest that both VR and YouTube learning platforms are useful in educational settings despite the differences between the level of student engagement and interaction.

Beyond knowledge outcomes, there is growing evidence that further supports the use of VR in broader geriatric education, such as in dementia care training. Broader literature indicates that training programs that use VR provide a risk-free environment to allow learners to hone these clinical skills and build confidence as well as empathy before applying them in real-world settings [17, 18]. A VR dementia training program called “myShoes” had used VR to place learners in the perspective of a person living with dementia and showed that participants afterwards better understood dementia symptoms and developed more empathy towards individuals with dementia [19]. These findings align with the principles of the 4Ms framework, such as the “What Matters” and “Mentation” concepts.

Strengths

This project contains some strengths worth noting. Larger and diverse sample sizes allowed the project to increase its reliability. In addition, our work was also able to highlight and compare both VR and YT platforms of digital learning, rather than focusing on the benefits of one platform.

Limitations and future research

Several limitations exist in this project and should be acknowledged. Participants self-selected their training modality, VR or YT. This introduces potential selection bias. While baseline pre-test scores were not statistically significant between training groups, there were several unmeasured factors, such as learning preferences and familiarity with the technology, that can influence the results.

There is also a possibility for response bias due to knowledge outcomes being based on self-reported perceived knowledge rather than an objective examination. Additionally, the final sample size decreased after excluding participants who did not completely fill out the survey. This is likely due to the survey sections being optional to complete. This study also lacked in assessing the long-term impact of the training due to a lack of follow-up in participants. Furthermore, results cannot be generalized as there was no comparison group on didactics; therefore, it is difficult to determine if one is better than the other.

Since both platforms yielded similar results, this creates opportunities for expanding VR and YouTube training to other geriatric-related interventions and quality improvement initiatives, which future research can further explore. It would also be beneficial for follow-up studies to focus on the long-term implications of the training or to apply the training to various institutions to widen the demographic profile.

## Conclusions

Both VR and YT platforms yield similar results and therefore create opportunities for expanding VR and YouTube training to other geriatric-related interventions and quality improvement initiatives, which future research can further explore. It would also be beneficial for follow-up studies to focus on the long-term implications of the training or to apply the training to various institutions to widen the demographic profile. Both VR and YT platforms significantly enhanced medical students’ understanding of the Age-Friendly Health System (AFHS) and the 4Ms framework. However, statistical comparisons did not reveal a significant difference between the two modalities, suggesting using both methods as learning tools in a medical academic setting. Future research can explore long-term knowledge retention and the application of both methods of learning in diverse institutions. Overall, VR and YouTube are beneficial in preparing students to be future physicians in delivering better quality of care for older adults.

## Appendices

Appendix 1

**Table 4:** GWEP Training Knowledge Survey Credits: Winnie Chen, Michelle Ngo, Sweta Tewary

Question #	Section	Item	Response Options
1	General Information	Date of completion	Open Response
2		Presentation date	MM/DD/YYYY
3		Site name	Open Response
4		Name of presenter	Open Response
5		Training topic	Virtual Reality case study on AFHS and 4Ms; Advance Care Planning; Cultural & Linguistic Competency; Dementia Care; Deprescribing/Appropriate Prescribing; Diabetes; Elder Abuse; Emergency Preparedness; End-of-Life Care; Eye Health; Fall Prevention; Healthcare & Financial Literacy; Heart Failure/Other Chronic Diseases; Infection Prevention; LGBTQ Care; Long-Term Care; Lung Disease; Mental Health; Nutrition; Opioids; Oral Health; Population Health; Reduce Hospitalizations/Improvements in Care; Rehabilitation & Long-term Care Placement; Social Determinants of Aging; Spirituality & Integrated Care; Telehealth; Value-based Care; Other (specify)
6		Was today’s activity part of a journal club?	Yes / No
7	Professional Background	Discipline	Audiology; Behavioral Health; Dentistry; Dietetics; Medicine; Nursing; Optometry; OT; Pharmacy; Psychiatry; PT; Public Health; Speech Pathology; Other
8		Role	Student; Resident; Fellow; Faculty/Administrator; Other
9	Pre/Post Training Knowledge	Before training, how much knowledge do you feel you have in this topic?	None; Low; Moderate; High
10		After training, how much knowledge do you feel you have in this topic?	None; Low; Moderate; High
11		Do you think you will apply what you learned in your role?	Very likely; Somewhat likely; Not likely
12		What would you like to know more about in future sessions?	Open Response
13		How can we improve this training for future students/professionals?	Open Response
14	Demographics	Age group	≤19; 20–29; 30–39; 40–49; 50–59; ≥60
15		Gender	Male; Female
16		Veteran status	Reservist; Veteran; No
17		Ethnicity	Hispanic/Latino; Not Hispanic/Latino
18		Race	White; Black/African American; Asian; American Indian/Alaska Native; Native Hawaiian/Pacific Islander
19		Rural residential background	Yes / No
20		Disadvantaged background	Yes / No
21		Underrepresented minority in your profession	Yes / No
22		Affiliation with CanoHealth	Yes / No
